# Knowledge, attitude and perceptions about Crimean Congo Haemorrhagic Fever (CCHF) among occupationally high-risk healthcare professionals of Pakistan

**DOI:** 10.1186/s12879-020-05714-z

**Published:** 2021-01-07

**Authors:** Ali Ahmed, Muhammad Saqlain, Maria Tanveer, Azhar Hussain Tahir, Fakhar Ud-Din, Maryum Ibrar Shinwari, Gul Majid Khan, Naveed Anwer

**Affiliations:** 1 School of Pharmacy, Monash University, Jalan Lagoon Selatan, Bandar Sunway, 47500 Subang Jaya, Selangor Malaysia; 2 Department of Pharmacy, Quaid-i-Azam University, Islamabad, 45320 Pakistan; 3 PASTIC National Centre, Quaid-e-Azam University Campus, Islamabad, Pakistan

**Keywords:** CCHF, Eid-ul-Adha, Healthcare workers, High risk, Zoonotic, Pakistan

## Abstract

**Background:**

Crimean Congo Haemorrhagic Fever (CCHF), a tropically neglected infectious disease caused by *Nairovirus*, is endemic in low middle-income countries like Pakistan. Emergency health care professionals (HCPs) are at risk of contracting nosocomial transmission of CCHF. We, therefore, aim to analyze the knowledge, attitudes, and perceptions (KAP) of at-risk physicians, nurses, and pharmacists in Pakistan and the factors associated with good KAP.

**Method:**

A validated questionnaire (Cronbach’s alpha 0.71) was used to collect data from HCPs in two CCHF endemic metropolitan cities of Pakistan by employing a cross-sectional study design. For data analysis percentages, chi-square test and Spearman correlation were applied by using SPSS version 22.

**Results:**

Of the 478 participants, 56% (*n* = 268) were physicians, 37.4% (*n* = 179) were nurses, and 6.5% (*n* = 31) were pharmacists. The proportion of HCPs with good knowledge, attitude, and perception scores was 54.3%, 81, and 69%, respectively. Being a physician, having more work experience, having a higher age, working in tertiary care settings, were key factors for higher knowledge (*p* < 0.001). The correlation coefficient showed significant positive correlation between attitude- perception (r = 0.560, *p* < 0.001).

**Conclusion:**

We have observed average knowledge of HCPs. Therefore, we recommend time to time education campaigns and workshops in highly endemic CCHF regions to be launched by health ministries and HCPs, in particular nurses, encouraged to follow authentic academic sources of information to prevent nosocomial transmission.

**Supplementary Information:**

The online version contains supplementary material available at 10.1186/s12879-020-05714-z.

## Background

Crimean Congo Haemorrhagic Fever (CCHF) or viral hemorrhagic fever is a zoonotic disease spread by *Hyalomma* tick bites or by contact with infected animals [1, 2]. The pleomorphic RNA virus causes CCHF belonged to the family *Nairoviridae* the genus *Orthonairovirus* [3]. This lethal virus is seroprevalent in the Middle East, Central African, and Asian countries such as India and Pakistan [4]. CCHF outbreaks pose a threat to public health systems due to its long-term and severe mechanism of infection, its propensity for epidemics, its high case-fatality rate (10–40%) and its capacity for nosocomial outbreaks, and its diagnosis and preventive difficulties [5–7].

CCHF virus transmission occurs mainly through the crushing of carrier ticks by bare hands, contact with infected blood or tissues of infected animals during or after slaughter, human to human transmission may also occur through contact with body fluids, blood or organs of the infected person [7–9]. Nosocomial infection may also result from contact with contaminated medical-surgical equipment, improper sterilization and re-use of the injection needles [6, 7, 10]. During Eid ul Adha, the largest Muslim religious festival in which millions of animals are slaughtered, the rate of new infections increase several times [11–13]. Farmers, dairy workers, slaughterhouse workers, veterinarians, and Health Care Professionals (HCPs) in endemic regions are at high risk of contracting CCHF [7, 10, 13].

Crimean Congo Hemorrhagic Fever Virus (CCHFV) continues to increase in Pakistan between January 2014 and May 2020, with around 356 CCHF patients confirmed across the country by the National Institute of Health, Islamabad, Pakistan, with a 25% mortality rate [14–16]. Of these patients, 38% were reported from Balochistan, followed by 23% from Punjab, 19% from Khyber Pakhtunkhwa, 14% from Sindh, and 6% from Islamabad [14, 16]. Zohaib et al. reported 2.7% of CCHF seroprevalence in Pakistan with increased prevalence in rural residents, possibly due to increased exposure to animals [17]. High risk of nosocomial transmission of CCHFV was reported for the first time in 1976 when laparotomy of a CCHF patient was performed in Pakistan, resulting in 11 secondary cases of hospital transmission leading to 3 deaths [10, 18]. Recently, due to the highly contagious nature of CCHF and not following serious precautionary measures, multiple nosocomial transmissions and deaths of HCPs have occurred in different regions of Pakistan, such as in Quetta, Abbottabad, Rawalpindi, and Bahawalpur [19–21]. HCPs should know with what signs CCHF patients will visit hospitals and be aware of the potential for imports of CCHF from endemic regions, human to human nosocomial transmission [10, 22]. Early suspicion and diagnosis are needed to take immediate and strict precautions to prevent transmission in the community and the hospital [23]. All HCPs that may be involved in CCHF case management should receive adequate education regarding disease [24].

Knowledge, Attitude, and Perception (KAP) studies provide information on existing programs, and their results help develop future effective behavioral strategies [25]. Some studies have reported KAP of students and HCPs about CCHF in Pakistan, Iran, and Turkey [10, 23, 26–28]. However, due to the limited evidence available, we aim to conduct knowledge, attitude, and perceptions assessment study among occupationally at-risk HCPs in the endemic region of Pakistan. The findings of the study will be essential to highlight the significant gaps in the literature on HCPs awareness of CCHF, which, in turn, will help in the design of appropriate interventions to prevent future nosocomial transmission.

## Methods

### Ethical approval

The study was performed in accordance with the declaration of Helsinki. Ethical approval was first obtained from the Department of Pharmacy, Quaid I Azam University, Islamabad (Letter No: QAU/PharmacyDept/214), and the other ethical permission was obtained from the Ethical Review Board of Shaheed Zulfiqar Ali Bhutto Medical University (Letter No: F.1–1/2016/ERB/SZABMU/08/16).

### Study design and settings

The multicenter, institutional-based, cross-sectional study was conducted in Islamabad and Rawalpindi, the two metropolitan cities of Pakistan. We choose these cities because previously, nosocomial transmissions of CCHF have been reported from these areas [18, 29, 30]. Islamabad and Rawalpindi are in the Potohar region of northern Punjab. The data was collected from five tertiary care government hospitals (Pakistan Institute of Medical Sciences, Islamabad; Polyclinic Hospital, Islamabad; Benazir Bhutto Hospital, Rawalpindi; Holy Family Hospital, Rawalpindi; District Health Quarter, Civil Hospital, Rawalpindi) and two private secondary care hospitals (Maroof International Hospital, Islamabad & Ali Medical Complex, Islamabad). The STROBE (Strengthening the reporting of observational studies in epidemiology) checklist was used to report the current analysis.

### Sample size, participants and procedure

The sample size calculated by Raosoft calculator was 377 by assuming 95% CI, 5% margin of error, Z of 1.96, and considering the 20,000 population [31]. To get more reliable results, we approached 563 HCPs. A convenient sampling technique was used to collect data from medical institutions. We targeted emergencies and infectious disease departments of the selected hospitals to enroll only those HCPs who have significant chances of exposure to CCHF. The study population included only mainstream HCPs such as physicians, pharmacists, and nurses working in hospitals. All HCPs working in other hospital departments such as dentistry, psychiatry, neurology, obstetrics, gynaecology, haematology, blood bank, endocrinology, nephrology, hepatology, and cardiology were excluded from the study. Students, laboratory staff, and administrative staff were also excluded from the study. The study was carried out from August 2018 to January 2019. Out of 563 questionnaires that were distributed in consented participants, 501 (88.9%) questionnaires were returned. The data was collected during working hours, and questionnaires were distributed and collected by the trained principal investigators.

### Study questionnaire

The study instrument was designed based on the thorough literature review and information retrieved from the official webpage of the World Health Organization (WHO) regarding CCHF transmission, precautions, epidemiology, seasonal impact, clinical management, and previous studies [23, 27, 32, 33]. The questionnaire was validated in two steps in the first step initial version was sent to researchers from academia, medical professionals (Physicians, Nurses, and Pharmacists) for their suggestions regarding appropriateness, conciseness, and importance. After amendments, in second phase instrument was pilot tested on conveniently available 45 HCPs, including the equal number of pharmacists, doctors, and nurses for cognitive debriefing. Participants were requested to provide feedback on any issues relating to the understanding of the questionnaire. Two questions were modified based on the comments of the participant. The reliability of the instrument was checked by SPSS Version 26, overall Cronbach’s alpha was reported to be 0.71, and the participants of the pilot study were not included in the final analysis.

The final version of the tool consisted of four parts for the assessment of demographics, knowledge, attitudes, and perceptions. The demographic section was comprised of gender, age, profession, experience, city of practice, and one item about the major source of information regarding CCHF. The knowledge section included 24 questions and assessed by giving a score of 1 to correct answer and 0 to the wrong answer. Items were evaluated in multiple-choice questions. If HCPs correctly answer 14 questions, he/she regarded as having good knowledge.

The attitude section had six questions, and each item was recorded on a 5-point Likert scale (1 strongly disagree, 2 disagree, 3 neutral, 4 agree, 5 strongly agree). Likewise, the perception portion had 7 questions, and each was assessed on 5 points Likert scale (1 strongly disagree, 2 disagree, 3 neutral, 4 agree, 5 strongly agree).

### Statistical analysis

The statistical analysis was performed by SPSS version 26. Frequencies and percentages have been calculated for categorical variables. Chi-square tests were used to investigate the statistically significant association between the demographic characteristics of the respondent and the KAP items. *p* < 0.05 was considered statistically significant. Spearman correlation tests were also performed to find a statistically significant correlation between knowledge, attitude, and perception scores.

## Results

A total of 478 participants were included in the final analysis after the removal of questionnaires containing missing information (*n* = 23). Table 1 shows demographics of the study population as 68.4% were female; 56.1% were physicians; 36% were 26–33 years of age; 58% were married, and 81.4% were working in tertiary care settings. For CCHF-related information, 20% of HCPs prefer research articles, 40% chose social media sites (Facebook, Twitter, YouTube, and Television), 20% seek workshops/conferences and 20% like to read brochures and newsletters.

**Table 1:** Demographics of the study population

	n (%)
**Gender**	
Female	327 (68.4)
Male	151 (31.6)
**Profession**	
Physician	268 (56.1)
Nurses	179 (37.4)
Pharmacist	31 (6.5)
**Age**	
18–25	142 (30)
26–33	172 (36)
34–41	83 (17.4)
42–49	51 (10.7)
50 and above	30 (6.3)
**Marital Status**	
Yes	279 (58.4)
No	199 (41.6)
**Experience (Years)**	
0–3	200 (41.8)
4–6	138 (28.9)
7–9	83 (17.4)
10 and above	57 (11.9)
**City of Practice**	
Rawalpindi	211 (44.2)
Islamabad	267 (55.8)
**Category of Practice**	
Secondary	89 (18.6)
Tertiary	389 (81.4)

The study found that 54.3% (*n* = 260) of HCPs had good knowledge. 82% (*n* = 392) respondents correctly answered that contact with infected *Hyalomma* ticks leads to the Transmission of CCHF, *n* = 350 (73.2%) HCPs correctly identified the causative agent of CCHF. 54% (n = 260) were aware of the best prophylactic measures against CCHF. 64% (*n* = 306) familiar with the symptoms of CCHF viral disease. 58% (*n* = 282) were mindful of standard treatment options available for CCHF. 47.2% (*n* = 226) were aware of the route of administration of anti-CCHF drug Ribavirin. 42.5% (*n* = 203) reported Balochistan is the most affected province of Pakistan. Findings revealed that knowledge status was significantly differed by age, gender, marital status, experience, and practice category (*p* < 0.001). Results demonstrated that physicians have the highest knowledge score (67.5%) followed by pharmacists (64.5%) and nurses (40%). While knowledge status was not significantly differed by region (*p* = 0.005) (Tables 2 and 3).

**Table 2:** Knowledge of HCPs towards CCHF

Serial No.	Question	Correct n (%)	Incorrect n (%)
1	CCHF is caused by?	398 (83.21)	80 (16.80)
2	The predominant symptoms associated with CCHF are:	306 (64.01)	172 (35.98)
3	CCHF can be transmitted through percutaneous contact?	340 (71.12)	138 (28.87)
4	The spread of CCHF occurs through	281 (58.78)	197 (41.21)
5	Contact with an infected vector can be a mode of transmission to human	392 (82.00)	86 (17.99)
6	Contact with human blood and body fluids can also be transmission source	63 (13.17)	415 (86.82)
7	Contact with animals cannot transfer CCHF	225 (47.07)	253 (52.92)
8	Most affected province of Pakistan?	203 (42.46)	275 (57.53)
9	Most affected months of the year?	99 (20.71)	379 (79.28)
10	The most common cause of hospital born Congo Infection?	343 (71.75)	135 (28.24)
11	CCHF is highly symptomatic in infected animals:	160 (33.47)	318 (66.52)
12	The mortality rate of CCHF in Pakistan?	242 (50.62)	236 (49.37)
13	What diagnostic options are available for CCHF?	259 (54.18)	219 (45.81)
14	Is the standard treatment option available for CCHF?	282 (58.99)	196 (41.00)
15	Best prophylactic measures against CCHF?	260 (54.39)	218 (45.60)
16	Is CCHF a zoonotic disease?	339 (70.92)	139 (29.07)
17	Is there any vaccine available for CCHF?	222 (46.44)	256 (53.55)
18	Can CCHF be transmitted via air and water?	179 (37.44)	299 (62.55)
19	Can CCHF be transferred through social contacts	270 (56.48)	208 (43.51)
20	Can CCHF be cured entirely with medicine?	327 (68.41)	151 (31.58)
21	Contact with feces, urine, and saliva of an infected person can cause CCHF?	309 (64.64)	169 (35.35)
22	Does avoiding mosquitoes bites prevents CCHF?	181 (37.86)	297 (62.13)
23	Is Ribavirin taken as orally?	226 (47.28)	252 (52.71)
24	The loading dose of Ribavirin taken for CCHF is:	125 (26.00)	402 (84.10)

**Table 3:** Association of knowledge with demographics

	Good Knowledge n (%)	Poor Knowledge n (%)	***P***-Value
**Total**	**260 (54.39)**	**218 (45.61)**	
**Gender**			
Female	184 (56.2)	143 (43.8)	
Male	76 (50.3)	75 (49.7)	
**Profession**			< 0.001
Physicians	181 (67.5)	87 (32.5)	
Nurses	71 (40)	108 (60)	
Pharmacists	20 (64.5)	11 (35.5)	
**Age**			< 0.001
18–25	60 (42.3)	82 (57.7)	
26–33	89 (51.7)	83 (48.3)	
31–38	57 (68.7)	26 (31.3)	
39–46	39 (76.5)	12 (23.5)	
47 and above	25 (83.3)	5 (16.7)	
**Marital status**			< 0.001
Married	151 (54.1)	128 (45.9)	
Single	106 (55.5)	85 (44.5)	
**Experience of practice**			< 0.001
0–3	80 (47)	90 (53)	
4–6	73 (52.9)	65 (47.1)	
7–9	64 (62)	39 (38)	
> 10	56 (83.5)	11 (16.5)	
**Region**			0.055
Rawalpindi	112 (53)	99 (47)	
Islamabad	146 (54.7)	121 (45.3)	
**Category of practice**			< 0.001
Secondary	40 (44.9)	49 (55.1)	
Tertiary	246 (63.2)	143 (36.8)	

Significant *p* value < 0.05.

Regarding attitudes, about half (48.54%, & 49.37%) of respondents were agreed that early diagnosis had a positive effect on CCHF treatment, and supportive care can be helpful for CCHF, respectively. The responses were significantly (*p* < 0.005) varied by age, profession, and experience. Similarly, 46.65% HCPs assumed that they are at risk of getting the infection, and 48.74% felt fear in dealing with CCHF patients. The responses were significantly differed by experience, profession, age, category of practice (*p* < 0.005) (Table 4).

**Table 4:** Attitude of HCPs towards CCHF and differences in responses by demographic characteristics

Attitude assessing questions	SD	D	N	A	SA	Gender	Profession	Age	Marital status	Experience of Practice	Region	Category of practice
Effect of early diagnosis on CCHF	30 (6.28)	35 (7.32)	64 (13.39)	232 (48.54)	117 (24.48)	.692	**< 0.001**	**< 0.001**	**.009**	**.007**	**< 0.001**	**< 0.001**
Is supportive care helpful for CCHF	15 (3.14)	38 (7.95)	83 (17.36)	236 (49.37)	106 (22.18)	.494	**.004**	**.015**	**< 0.001**	**.006**	**< 0.001**	**< 0.001**
Are you at risk of contracting CCHF?	14 (2.93)	48 (10.04)	71 (14.85)	223 (46.65)	122 (25.52)	.282	**< 0.001**	**< 0.001**	**< 0.001**	**< 0.001**	**< 0.001**	**< 0.001**
Do you feel concerned while dealing with infected individuals?	22 (4.60)	51 (10.67)	112 (23.4)	233 (48.74)	60 (12.55)	.930	**.005**	**.004**	.144	**.049**	**< 0.001**	.119
Is the Health care system adequately equipped?	36 (7.53)	127 (26.6)	96 (20.08)	194 (40.59)	25 (5.23)	.619	**< 0.001**	**.002**	**< 0.001**	**.002**	**< 0.001**	**< 0.001**
Should there be a separate room for CCHF confirmed patient?	14 (2.93)	35 (7.32)	68 (14.23)	218 (45.61)	143 (24.48)	.331	**.028**	**< 0.001**	**< 0.001**	**.001**	**< 0.001**	**< 0.001**

*Abbreviations***:** *SD* strongly disagree, *D* Disagree, *N* Neutral, *A* Agree, *SA* Strongly agree.

**Note:** Bold values shown significant association

56% HCPs agreed that following standard procedures will minimize the risk of Transmission. The response was significantly differed by profession (*p* = 0.001). 48 and 51% HCPs agreed that those people who have pets at home and animal handlers are at increased risk of contracting the CCHFV. The responses varied significantly in terms of age, marital status, and practice experience (*p* < 0.05). 58% of healthcare professionals agreed that they were well equipped with the necessary quarantine observation skills. The response was significantly varied by profession (*p* < 0.001), marital status (*p* < 0.001), and experience (*p* = 0.01) (Table 5).

**Table 5:** Perception of HCPs towards CCHF and differences in responses by demographic characteristics

Perception assessing questions	SD	D	N	A	SA	Gender	Profession	Age	Marital status	Experience of Practice	Region	Category of practice
Will you follow standard procedures to minimize the risk of transmission of infection?	22 (4.60)	49 (10.25)	85 (17.78)	268 (56.07)	54 (11.30)	.273	**.001**	.053	.069	**.045**	.511	**< 0.001**
Do you have the quarantine skills that you need to observe?	32 (6.69)	67 (14.02)	96 (20.08)	250 (52.30)	33 (6.90)	**.048**	**< 0.001**	.051	**< 0.001**	**.010**	.169	**< 0.001**
Use of preventive medicines while dealing with CCHF patients?	23 (4.81)	83 (17.36)	96 (20.08)	238 (49.79)	38 (7.95)	.259	**.009**	**.002**	**< 0.001**	**.002**	.642	**< 0.001**
Do you have valuable sources of information for CCHF?	20 (4.18)	75 (15.69)	92 (19.25)	258 (53.97)	33 (6.90)	**.006**	**.001**	**< 0.001**	**< 0.001**	**< 0.001**	.452	**< 0.001**
All healthcare professionals should go for mandatory CCHF testing during sporadic outbreaks	11 (2.30)	36 (7.53)	250 (52.3)	155 (32.43)	26 (5.44)	**.010**	**< 0.001**	**.001**	**.010**	**< 0.001**	**< 0.001**	**< 0.001**
Do pets increase the risk of CCHF?	17 (3.56)	54 (11.30)	97 (20.29)	231 (48.33)	79 (16.53)	.222	.110	**< 0.001**	**< 0.001**	**< 0.001**	.579	**< 0.001**
Animal herders are at additional risk of contracting disease	22 (4.60)	28 (5.86)	75 (15.69)	245 (51.26)	108 (22.6)	.539	**.005**	**.001**	**.004**	**.021**	.582	**< 0.001**

*Abbreviations***:** *SD* strongly disagree, *D* Disagree, *N* Neutral, *A* Agree, *SA* Strongly agree.

**Note**: Bold values shown significant association

Spearman correlation tests revealed that there is a strong linear positive correlation between attitude-perception (r = 0.560, *p* < 0.001). However, there is a weak positive correlation between knowledge-attitude (r = 0.092, *p* = 0.045).

## Discussion

To effectively treat CCHF patients without risk of nosocomial infection, HCPs need to have good knowledge, attitude, and practices of preventive measures, such as before visiting a patient, wearing gloves, masks, protective clothing, goggles, disposable clothing and face shields [33]. Very little was found in the literature on the question of emergency HCPs’ knowledge attitude and perceptions, so this is the first study in the CCHF endemic region of Pakistan where previously frequent nosocomial transmissions have been reported [12, 18, 29, 34].

Our study reported 54.3% HCPs to have good knowledge while Rahnavardi et al. reported 50.34% of participants had good knowledge [23]. A possible explanation of higher knowledge in our study might be that Iranian research has included a smaller number of physicians and nurses while included more paramedics, laboratory personals, and orderlies [23]. However, in the case of senior physicians, Iran and the Turkish study reported a higher proportion of 90.20 and 84.27% than our 83% [23, 35]. Our study reported better knowledge then the survey conducted among medical and pharmacy students in Pakistan [27]. One possible explanation might be that students are more focused on their current subjects for examination, while HCPs in our study deal directly with patients so that they are better prepared.

Of note that, according to an Iranian study when the question was asked; CCHF can be transmitted through percutaneous contact from an infected individual, 89.5% participants provided correct answer while in our study 71% study participants provided correct answers similarly in Turkish study 98.2% physicians correctly answer this question [23, 36]. This finding is of particular interest as the percutaneous route of nosocomial Transmission has been identified as a potential route for CCHF transmission in the past nosocomial outbreak of CCHF in Rawalpindi, Pakistan [18]. In our study, only 21 and 42% of HCPs were aware of the most affected months, and the most affected province of Pakistan from CCHF, a considerable lack of epidemiological knowledge, was observed. Since the disease is endemic in Pakistan and the study area is at risk of illness due to the regular and more frequent transport of livestock during the Eid ul Adha season [12], it is vital to train health workers and to distribute brochures and posters containing CCHF-related information to all health clinics and health centres.

In 2000, Sheik et al. indicated that there was a lack of knowledge of HCPs regarding prevention and burial procedures for CCHF patients [26]. Our results have shown a significant association between higher education, experience, and higher knowledge, a finding that is consistent with that of Turkish and Iranian studies [23, 35, 36]. Those working in tertiary care settings have more knowledge than those working in secondary care settings, possibly due to more facilities, workshops, and patient flow, and these results are consistent with the study conducted in India [37].

In Pakistan, nurses are at the highest risk because they are the first HCPs to interact with patients for passing the Intravenous line and taking blood samples for testing, and there is a chance of accidental needle stick injury that could result in nosocomial Transmission [38]. Educational training should be provided to nurses to educate them about protective measures and, if they have received such injury, what they should do immediately as they should know about the dose of Ribavirin (antiviral) to be used as prophylaxis.

Since emergency HCPs frequently encounter CCHF patients and have a high chance of percutaneous infection, 46% of HCPs in our study believe that Pakistan’s health will not be able to cope appropriately if such an incident occurs in the same way as Haq et al. reported the incompetence of Pakistan’s healthcare system in dealing with health emergencies [39]. The majority of study subjects agreed that animal herders and having pets increased risk of CCHF infection, just like Georgian and Turkish studies [8, 40, 41].

Despite the positive attitudes and perceptions of HCPs towards CCHF, knowledge of HCPs about CCHF was poor. Possible explanations may be due to a busy schedule and additional workload, as well as a lack of regular education programs. HCPs continuing education in CCHF should be used to raise awareness of CCHF preventive methods, treatment options, identification of ineffective diagnostic factors, the role of *hyalomma* ticks in Transmission, disease epidemiology, most affected months of the year, and any specific event that may result in CCHF transmission, such as Eid ul Adha. Senior physicians, despite having higher knowledge, could not motivate junior physicians, nurses, pharmacists to improve their knowledge and practice. Despite their relative higher education, physicians (especially senior), who could have motivated other HCPs toward better attitudes and safer practices, did not prove to have the best attitudes themselves.

Decades of research has shown that people have the capacity to voluntarily forget things, so to keep things in mind, reminders of information are needed [42]. Reading the authentic source of information is also essential to obtain the correct information so that the government and hospital policymakers should guide the recommended sources of information such as the WHO and the Center for Disease Control (CDC) guidelines. The Government of Pakistan and the higher authorities of the hospital should organize workshops and seminars periodically to keep hospital staff well informed about such neglected tropical diseases, especially before their peak seasons [36, 37]. HCPs should be ensured to follow the standard safety protocols, and hospital administration should ensure the availability of quarantine facilities for CCHF patients in hospitals. Further research is needed to evaluate the practices of HCPs concerning the following guidelines for preventive measures and safe disposal of syringes, medical devices and reporting and managing percutaneous exposures. Further research should also be conducted to assess the seroprevalence of CCHF in HCPs.

### Strength and limitations

It is the first large-scale study in the endemic CCHF region of Pakistan, where there is not much literature available. The results of this study are useful for stakeholders and health policy officials to develop culturally appropriate interventions. The research identified differences in the necessary information and practice areas that could be addressed in future educational and learning activities. The findings also demonstrated that HCPs used less authentic sources of information; this should be addressed immediately as it ultimately affects knowledge and is reflected in attitudes and practices.

Research has some drawbacks, such as the use of a questionnaire to assess KAP, which can result in recall bias or social credibility bias. We recruit only HCPs working in emergencies of hospitals and infectious disease department staff, so the findings of the study can’t be generalized for all categories of HCPs. CCHF is endemic in rural areas, while we have only collected data from hospitals in metropolitan areas, so caution should be taken when interpreting findings for rural settings.

## Conclusion

Overall knowledge, attitude, and perceptions of high-risk HCPs reported in our study are lower than expectations. There is a serious need to arrange occupational safety and education courses for all HCPs working in endemic regions of Pakistan to encourage them to use only authentic academic sources of information. In the meantime, special attention is paid to nurses and less experienced HCPs to enhance their knowledge.

## Supplementary Information

**Additional file 1.**

## Data Availability

Data is available from the corresponding author on a reasonable request.

## Abbreviations

CCHF: Crimean Congo Haemorrhagic Fever
CCHFV: Crimean Congo Hemorrhagic Fever Virus
HCPs: Health Care Professionals
KAP: Knowledge, Attitudes, and Perceptions
WHO: World Health Organization
