# Supplementary material for: Knowledge, attitude and perceptions about Crimean Congo Haemorrhagic Fever (CCHF) among occupationally high-risk healthcare professionals of Pakistan
Source: BMC Infect Dis. 2021 Jan 7;21:35. doi: 10.1186/s12879-020-05714-z (PMC7792042; doi:10.1186/s12879-020-05714-z)
Supplement: Supplementary file 1 — Additional file 1. [file 12879_2020_5714_MOESM1_ESM.docx]

**Questionnaire used for data collection**

**Demographics Questions:**

1. Name (Optional):

2. Gender: F ☐ M ☐

3. Age (Years): 18-25 ☐ 26-33 ☐ 34-41 ☐ 42-49 ☐ 50 and above ☐

4. City of practice: Islamabad ☐ Rawalpindi ☐

5. Experience (Years): 0-3 ☐ 4-6 ☐ 7-9 ☐ 10 and above ☐

6. Category of practice: Secondary care ☐ Tertiary care ☐

8. Marital status: Yes ☐ No ☐

**Knowledge indicator questions:**

1. What do you know about the causing factor of CCHF?

Fungi ☐ Bacteria ☐ Virus ☐ No Idea ☐

2. The spread of CCHF occurs through:

Mosquito☐ Ixodid hard ticks☐ Cat fleas☐ Infected flies☐

3. Contact with an infected vector can be a mode of transmission to a human:

Yes☐ No☐ No Idea ☐

4. Contact with infected human blood and body fluids can also be transmission source:

Yes☐ No☐ No Idea ☐

5. Contact with animals cannot transfer CCHF:

Yes☐ No☐ No Idea ☐

6. Most affected province of Pakistan?

Punjab ☐ Sindh ☐ Balochistan☐ KPK ☐ No Idea ☐

7. Most affected months of the year?

Jan-Mar ☐ Jun-Aug ☐ Sept-Nov ☐

Same throughout the year ☐ No Idea ☐

8. CCHF can be transmitted through percutaneous contact?

Yes☐ No☐ No Idea ☐

9. The most common cause of hospital born Congo infection?

Use of unsterilized medical equipment ☐

Percutaneous infection to laboratory personnel ☐

Poor infection control practices ☐ All of the above ☐

10. The predominant symptoms associated with CCHF are:

Hemorrhage and fever☐ Headache and Joint pain☐

Generalized red spots☐ all of the above☐

11. CCHF is highly symptomatic in infected animals:

Yes☐ No☐ No Idea ☐

12. The mortality rate of CCHF in Pakistan?

Extremely high fatality rate (40-60% ☐ High Fatality rate (10-40%) ☐

Medium Fatality rate (10-20%) ☐ Low Fatality rate (5%) ☐

13. What is the diagnostic option (s) available for CCHF?

ELISA (Enzyme-linked Immunosorbent assay)☐

RT-PCR (real-time polymerase chain reaction)☐

Combination of ELISA and RT-PCR ☐All of the above ☐

14. Select the standard treatment option available for CCHF?

Ribavirin ☐Fidaxomicin ☐Ceftaroline ☐

Caspofungin ☐No Idea ☐

15. Select the best prophylactic measure(s) against CCHF among the following?

Using DEET insect repellent on exposed skin and clothing ☐

Burning the dead bodies of infected animals ☐

Proper disposal of infectious human blood from hospitals☐

All of the above ☐

16. Is CCHF a zoonotic disease?

Yes ☐ No ☐ No Idea ☐

17. Can CCHF be transmitted via air and water?

Yes ☐ No ☐ No Idea ☐

18. Can CCHF be transferred through social contacts like sharing clothes, Cups/ plates/ spoon/ glass, bathrooms, shaking hands & kissing?

Yes ☐ No ☐ No Idea ☐

19. Can CCHF be cured entirely with medicine?

Yes ☐ No ☐ No Idea ☐

20. Contact with feces, urine, and saliva of an infected person can cause CCHF?

Yes ☐ No ☐ No Idea ☐

21. Does avoiding mosquitoes bites prevents CCHF?

Yes ☐ No ☐ No Idea ☐

22. Is Ribavirin taken as orally?

Yes ☐ No ☐ No Idea ☐

23. The loading dose of Ribavirin taken for CCHF is:

2g☐ 4g☐ 6g☐ No Idea ☐

24. Is there any vaccine available for CCHF?

Yes ☐ No ☐ No Idea ☐

**Attitude indicators questions:**

1. Do you think early diagnosis can lead to the rapid resolution of symptoms of hemorrhagic fever?

Strongly Disagree☐ Disagree ☐ Neutral ☐Agree ☐ strongly agree ☐

2. Do you think the severity of the disease can be decreased through the management of electrolyte and fluid imbalance and the provision of supportive care through blood, plasma, and platelet replacement?

Strongly Disagree☐ Disagree ☐ Neutral ☐ Agree ☐ strongly agree ☐

3. Do you think the lack of effectively isolated building facilities poses a significant risk to health professionals dealing with infected individuals?

Strongly Disagree☐ Disagree ☐ Neutral ☐Agree ☐ strongly agree ☐

4. Keeping in view the contagious nature of the infectious agent, do you think you will feel concerned in dealing with infected individuals?

Strongly Disagree☐ Disagree ☐ Neutral ☐ Agree ☐strongly agree ☐

5. Do you think the health care system is adequately equipped to provide isolated body fluid collection and testing setup for Congo infected individuals?

Strongly Disagree☐ Disagree ☐ Neutral ☐ Agree ☐strongly agree ☐

6. Do you think there should be a separate room for CCHF confirmed patient?

Strongly Disagree☐ Disagree ☐ Neutral ☐ Agree ☐ strongly agree ☐

**Perceptions assessing questions:**

1. Do you take standard blood testing procedures to minimize the risk of transmission of infection?

Strongly Disagree ☐Disagree ☐ Neutral ☐ Agree ☐ strongly agree ☐

2. Are you equipped with isolation observing skills necessary to protect yourself from contracting the disease while working with infected individuals?

Strongly Disagree☐ Disagree ☐ Neutral ☐ Agree ☐ strongly agree ☐

3. Do you use preventive medicines when dealing with patients suffering from highly contagious diseases?

Strongly Disagree☐ Disagree ☐ Neutral ☐ Agree ☐ strongly agree ☐

4. Do you consider you have a valuable source of information to look up to in case of confusion about dealing with infected individuals?

Strongly Disagree☐ Disagree ☐ Neutral ☐ Agree ☐ strongly agree ☐

5. In my opinion, all healthcare students and professionals should go for mandatory CCHF testing during extreme outbreaks:

Strongly Disagree ☐ Disagree☐ Neutral ☐ Agree ☐ strongly agree ☐

6. Do you think having birds and animals at your home can put you at additional risk for CCHF?

Strongly Disagree☐ Disagree ☐ Neutral ☐Agree ☐ strongly agree ☐

7. Herders of animals, individuals, working with livestock, and slaughterhouse workers are at a higher risk of CCHF infection?

Strongly Disagree☐ Disagree ☐ Neutral ☐ Agree ☐strongly agree ☐

**Signature..................**
